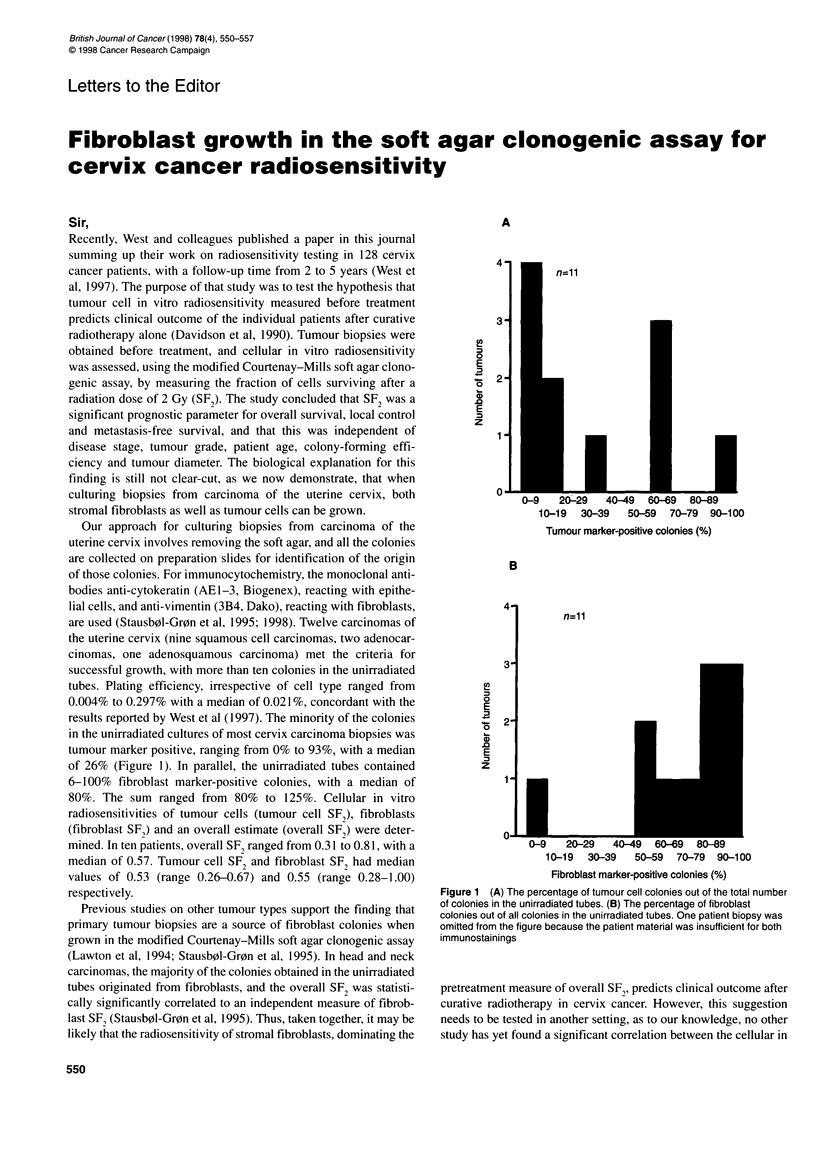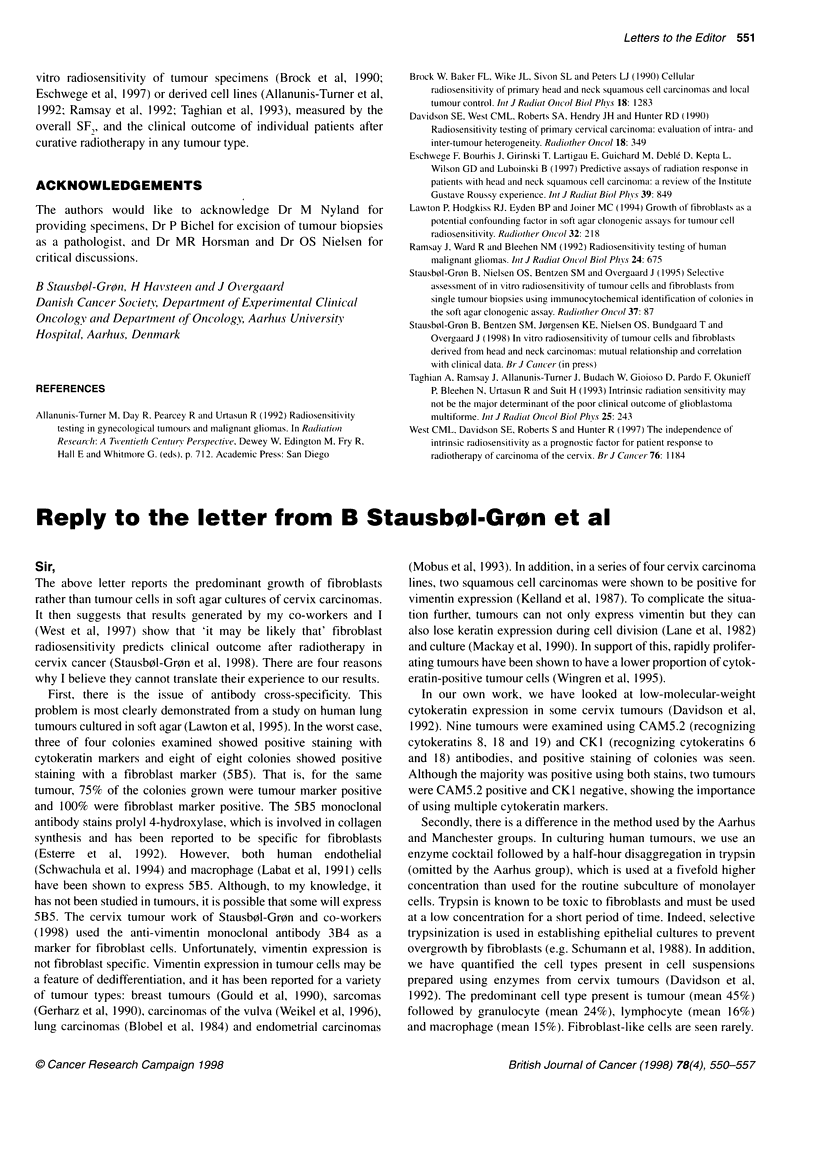# Fibroblast growth in the soft agar clonogenic assay for cervix cancer radiosensitivity.

**DOI:** 10.1038/bjc.1998.531

**Published:** 1998-08

**Authors:** B. Stausbøl-Grøn, H. Havsteen, J. Overgaard


British Journal of Cancer (1998) 78(4), 550–557
© 1998 Cancer Research Campaign

Letters to the Editor

### Fibroblast growth in the soft agar clonogenic assay for cervix cancer radiosensitivity

**Sir,**

Recently, West and colleagues published a paper in this journal summing up their work on radiosensitivity testing in 128 cervix cancer patients, with a follow-up time from 2 to 5 years (West et al, 1997). The purpose of that study was to test the hypothesis that tumour cell in vitro radiosensitivity measured before treatment predicts clinical outcome of the individual patients after curative radiotherapy alone (Davidson et al, 1990). Tumour biopsies were obtained before treatment, and cellular in vitro radiosensitivity was assessed, using the modified Courtenay–Mills soft agar clonogenic assay, by measuring the fraction of cells surviving after a radiation dose of 2 Gy ($SF_2$). The study concluded that $SF_2$ was a significant prognostic parameter for overall survival, local control and metastasis-free survival, and that this was independent of disease stage, tumour grade, patient age, colony-forming efficiency and tumour diameter. The biological explanation for this finding is still not clear-cut, as we now demonstrate, that when culturing biopsies from carcinoma of the uterine cervix, both stromal fibroblasts as well as tumour cells can be grown.

Our approach for culturing biopsies from carcinoma of the uterine cervix involves removing the soft agar, and all the colonies are collected on preparation slides for identification of the origin of those colonies. For immunocytochemistry, the monoclonal antibodies anti-cytokeratin (AE1–3, Biogenex), reacting with epithelial cells, and anti-vimentin (3B4, Dako), reacting with fibroblasts, are used (Stausbøl-Grøn et al, 1995; 1998). Twelve carcinomas of the uterine cervix (nine squamous cell carcinomas, two adenocarcinomas, one adenosquamous carcinoma) met the criteria for successful growth, with more than ten colonies in the unirradiated tubes. Plating efficiency, irrespective of cell type ranged from 0.004% to 0.297% with a median of 0.021%, concordant with the results reported by West et al (1997). The minority of the colonies in the unirradiated cultures of most cervix carcinoma biopsies was tumour marker positive, ranging from 0% to 93%, with a median of 26% (Figure 1). In parallel, the unirradiated tubes contained 6–100% fibroblast marker-positive colonies, with a median of 80%. The sum ranged from 80% to 125%. Cellular in vitro radiosensitivities of tumour cells (tumour cell $SF_2$), fibroblasts (fibroblast $SF_2$) and an overall estimate (overall $SF_2$) were determined. In ten patients, overall $SF_2$ ranged from 0.31 to 0.81, with a median of 0.57. Tumour cell $SF_2$ and fibroblast $SF_2$ had median values of 0.53 (range 0.26–0.67) and 0.55 (range 0.28–1.00) respectively.

Previous studies on other tumour types support the finding that primary tumour biopsies are a source of fibroblast colonies when grown in the modified Courtenay–Mills soft agar clonogenic assay (Lawton et al, 1994; Stausbøl-Grøn et al, 1995). In head and neck carcinomas, the majority of the colonies obtained in the unirradiated tubes originated from fibroblasts, and the overall $SF_2$ was statistically significantly correlated to an independent measure of fibroblast $SF_2$ (Stausbøl-Grøn et al, 1995). Thus, taken together, it may be likely that the radiosensitivity of stromal fibroblasts, dominating the

**Figure 1** (**A**) The percentage of tumour cell colonies out of the total number of colonies in the unirradiated tubes. (**B**) The percentage of fibroblast colonies out of all colonies in the unirradiated tubes. One patient biopsy was omitted from the figure because the patient material was insufficient for both immunostainings pretreatment measure of overall $SF_2$, predicts clinical outcome after curative radiotherapy in cervix cancer. However, this suggestion needs to be tested in another setting, as to our knowledge, no other study has yet found a significant correlation between the cellular in vitro radiosensitivity of tumour specimens (Brock et al, 1990; Eschwege et al, 1997) or derived cell lines (Allanunis-Turner et al, 1992; Ramsay et al, 1992; Taghian et al, 1993), measured by the overall $SF_2$, and the clinical outcome of individual patients after curative radiotherapy in any tumour type.

### ACKNOWLEDGEMENTS

The authors would like to acknowledge Dr M Nyland for providing specimens, Dr P Bichel for excision of tumour biopsies as a pathologist, and Dr MR Horsman and Dr OS Nielsen for critical discussions.

*B Stausbøl-Grøn, H Havsteen and J Overgaard*

*Danish Cancer Society, Department of Experimental Clinical Oncology and Department of Oncology, Aarhus University Hospital, Aarhus, Denmark*

#### REFERENCES

Allanunis-Turner M, Day R, Pearcey R and Urtasun R (1992) Radiosensitivity testing in gynecological tumours and malignant gliomas. In *Radiation Research: A Twentieth Century Perspective*, Dewey W, Edington M, Fry R, Hall E and Whitmore G. (eds), p. 712. Academic Press: San Diego

Brock W, Baker FL, Wike JL, Sivon SL and Peters LJ (1990) Cellular radiosensitivity of primary head and neck squamous cell carcinomas and local tumour control. *Int J Radiat Oncol Biol Phys* **18**: 1283

Davidson SE, West CML, Roberts SA, Hendry JH and Hunter RD (1990) Radiosensitivity testing of primary cervical carcinoma: evaluation of intra- and inter-tumour heterogeneity. *Radiother Oncol* **18**: 349

Eschwege F, Bourhis J, Girinski T, Lartigau E, Guichard M, Deblé D, Kepta L, Wilson GD and Luboinski B (1997) Predictive assays of radiation response in patients with head and neck squamous cell carcinoma: a review of the Institute Gustave Roussy experience. *Int J Radiat Biol Phys* **39**: 849

Lawton P, Hodgkiss RJ, Eyden BP and Joiner MC (1994) Growth of fibroblasts as a potential confounding factor in soft agar clonogenic assays for tumour cell radiosensitivity. *Radiother Oncol* **32**: 218

Ramsay J, Ward R and Bleehen NM (1992) Radiosensitivity testing of human malignant gliomas. *Int J Radiat Oncol Biol Phys* **24**: 675

Stausbøl-Grøn B, Nielsen OS, Bentzen SM and Overgaard J (1995) Selective assessment of in vitro radiosensitivity of tumour cells and fibroblasts from single tumour biopsies using immunocytochemical identification of colonies in the soft agar clonogenic assay. *Radiother Oncol* **37**: 87

Stausbøl-Grøn B, Bentzen SM, Jørgensen KE, Nielsen OS, Bundgaard T and Overgaard J (1998) In vitro radiosensitivity of tumour cells and fibroblasts derived from head and neck carcinomas: mutual relationship and correlation with clinical data. *Br J Cancer* (in press)

Taghian A, Ramsay J, Allanunis-Turner J, Budach W, Gioioso D, Pardo F, Okunieff P, Bleehen N, Urtasun R and Suit H (1993) Intrinsic radiation sensitivity may not be the major determinant of the poor clinical outcome of glioblastoma multiforme. *Int J Radiat Oncol Biol Phys* **25**: 243

West CML, Davidson SE, Roberts S and Hunter R (1997) The independence of intrinsic radiosensitivity as a prognostic factor for patient response to radiotherapy of carcinoma of the cervix. *Br J Cancer* **76**: 1184